# The prostatic inflammation score and risk of biochemical recurrence following radical prostatectomy

**DOI:** 10.1007/s00345-025-05977-8

**Published:** 2025-10-09

**Authors:** Francesco Guzzi, Ugo Giovanni Falagario, Angelo Cormio, Antonella Ninivaggi, Marco Finati, Nicola d’Altilia, Antonio Fanelli, Ruggiero Antonio Fiorella, Gian Maria Busetto, Carlo Bettocchi, Daniele Castellani, Vincenza Conteduca, Giuseppe Carrieri, Francesca Sanguedolce, Luigi Cormio

**Affiliations:** 1 Department of Urology and Renal Transplantation, Policlinico Foggia, University of Foggia, 71122 Foggia, Italy; 2 Department of Molecular Medicine and Surgery, (Solna), Karolinska Institutet, Stockholm, Sweden; 3 Urology Unit, Azienda Ospedaliero-Universitaria delle Macrhe Università Politecnica Delle Marche, Via Conca 71, 60126 Ancona, Italy; 4 Department of Urology, Comprehensive Cancer Center, Medical University of Vienna, Vienna, Austria; 5 Department of Urology, Bonomo Teaching Hospital, 76123 Andria, Italy; 6 Department of Oncology, Policlinico Foggia, University of Foggia, 71122 Foggia, Italy; 7 Pathology Unit, Policlinico Foggia, University of Foggia, 71122 Foggia, Italy

**Keywords:** Prostate inflammation, Biochemical recurrence, Prostate cancer, Radical prostatectomy, Robotic surgery

## Abstract

**Background:**

Inflammatory processes are thought to contribute to the development and progression of prostate cancer (PCa). This study aimed to evaluate whether a novel Prostatic Inflammation Score (PIS), integrating Irani Grade (G) and Aggressiveness (A), could predict the risk of biochemical recurrence (BCR) following radical prostatectomy (RP).

**Methods:**

Patients undergoing biopsy and RP between 2013 and 2023 were included. PIS was assessed on hematoxylin and eosin-stained biopsy cores: PIS 1 (G 0–1/A 0–1), PIS 2 (G 2–3/A 0–1), and PIS 3 (G 0–3/A 2–3). BCR, considered as two consecutive PSA readings of ≥ 0.2 ng/mL, was evaluated through multivariable Cox regression analysis.

**Results:**

Among 679 patients (median follow-up: 36 months), 127 (18.7%) experienced BCR. Over a median follow-up of 36 months, 127 (18.7%) patients experienced BCR. BCR incidence at six years was highest in PIS 1 (30%) vs. PIS 2 (10%) and PIS 3 (9%) (*p* = 0.002). Compared to PIS 1, hazard ratios (HR) for BCR were 0.44 (95% CI: 0.27–0.74) for PIS 2 and 0.37 (95% CI: 0.15–0.91) for PIS 3. IRANI G scores inversely correlated with BCR risk (HR = 0.44, 95% CI: 0.27–0.74), while IRANI A scores (2–3) showed a nonsignificant trend (HR = 0.41, 95% CI: 0.17–1.02, *p* = 0.054). Gleason grade and pT stage were significantly associated with BCR, while margin status and pN stage were not.

**Conclusions:**

PIS 1 was found to be associated with BCR after RP. Should these findings be externally validated, PIS could represent a readily available inexpensive tool to predict BCR after RP.

## Introduction

Persistent inflammation is now acknowledged as a critical factor in the early stages and advancement of various cancer. The production of reactive oxygen species by immune cells during inflammation can result in DNA alterations that promote oncogenesis [1]. The inflammatory microenvironment is known to facilitate tumor cell migration, invasion, and the metastatic process in several malignancies [2, 3]. On the other hand, inflammation can also contribute to tumor immunosurveillance by enabling the immune system to detect and eliminate aberrant cells [4].

The relationship between prostatic inflammation (PI) and prostate cancer (PCa) is also quite controversial. Some studies postulate that the “inflammatory storm” may promote PCa while others point at a strict correlation between PI and development and progression of benign prostatic hyperplasia [5, 6]. In any case, PI is a common finding in prostate biopsy (PBx) cores, and its potential clinical relevance is underlined by current EAU guidelines on prostate cancer (PCa) recommending to report the presence/absence and type of inflammation (acute vs. granulomatous) in PBx cores [7].

Although various methods, including immunohistochemistry and molecular analyses, have been proposed to assess prostatic inflammation, these are typically expensive and unsuitable for standard practice [8, 9]. In contrast, the Irani score offers a clear, validated method to classify prostatic inflammation during routine hematoxylin and eosin pathology review, based on the extent and intensity of inflammatory infiltrates [10]. Specifically, the Irani Grade (G) depicts the extent of stromal infiltration by the inflammatory cells whereas the Irani Aggressiveness (A) depicts the extent of glandular infiltration.

High Irani A scores have been found to be associated with increased PSA levels due to rupture of the prostate epithelium [10], whereas high Irani G scores have been found to be associated with a greater risk of being diagnosed with benign prostatic obstruction (BPO) rather than PCa at PBx [11–13]. Low Irani G and low Irani A scores, conversely, have been found to be associated with an increased risk of PCa diagnosis on PBx [11]. Moreover, we previously demonstrated that low bioptic Irani G score predicted adverse pathology at radical prostatectomy (RP) in patients with low grade PCa [14]. While encouraging, this initial experience also pointed out that the presence of two different scores (G and A) makes difficult to interpret the results, particularly when they are heterogeneous. To overcome this problem, we combined Irani G and A scores into a single comprehensive prostatic inflammation score (PIS) which indeed was more effective in defining the association between stromal and glandular inflammation with the risk of being diagnosed with PCa at PBx [15]. In the present study we tested the ability of the novel PIS in predicting biochemical recurrence (BCR) in patients having undergone RP.

## Materials and methods

We analyzed data from consecutive patients who had PBx and RP at our institution between January 2013 and December 2023. Patients under 5-alpha reductase inhibitors (5-ARIs), patients who had already undergone surgical treatment for benign prostatic hyperplasia, patients with indwelling urethral catheters and those with serum PSA > 20 ng/mL were excluded. **Conversely**,** diabetic patients were not excluded from the study.** Prostate biopsies were carried out under local topical anesthesia, employing a standardized 18-core sampling approach under TRUS guidance (BK Medical Flex Focus 500) with an 18-gauge/25 cm biopsy needle (Bard Max-Core).[16–18] In patients with positive MRI findings, 3–5 targeted cores were obtained per suspicious lesion using an MRI-US fusion biopsy system. A retropubic open or a robot-assisted approach was used for RP, with pelvic lymph node dissection (PLND) being performed if Briganti score was higher than the suggested cut-offs. Patients were followed up every 3 months during the first year and every 6 months thereafter. PSA levels were measured at each follow-up visit. Biochemical recurrence (BCR) was defined as two consecutive PSA measurements ≥ 0.2 ng/mL. The study protocol was approved by the University of Foggia Ethics Committee (Decision No. 152/CE/2014).

### Pathological analysis

**Prostate biopsy specimens** were analyzed by two experienced genitourinary pathologists following current ISUP guidelines. They utilized a 4-point scale to classify both Irani G and Irani A, **and had for long time done this together to improve their intra- and inter-observer performance.** Irani G was categorized as follows (0 - no inflammatory cells present, 1 – scattered inflammatory cell infiltrate, 2 - nonconfluent lymphoid nodules, and 3 – extensive inflammatory areas with confluent infiltrates. Irani A was defined (0 - no contact between inflammatory cells and glandular epithelium, 1 - contact between inflammatory cell infiltrates and glandular epithelium, 2 - evident but limited glandular epithelium disruption affecting less than 25% of the examined material, and 3 - glandular epithelium disruption involving more than 25% of the examined material).

### Statistical analysis

Continuous variables were reported as median values and interquartile ranges (IQR) and compared using the ANOVA and Kruskal-Wallis tests. Categorical variables were reported as percentages and tested using the Chi-square test. The association between the novel PIS and the diagnosis of any PCa and clinically significant prostate cancer (csPCa) defined as ISUP Gleason Grade Group (GGG) ≥ 2 was tested using multivariable logistic regression models, adjusting for age, PSA levels, biopsy history, and prostate volume. The same analyses were carried out for the original Irani G and A scores. In patients having undergone RP, the association between the novel PIS and BCR was tested using a multivariable Cox regression model incorporating PIS, adjusting for covariates known to be associated with BCR (age, PSA, pGGG, pT stage, margin status, and pN stage). The inverse Kaplan-Meier method was used to compute the cumulative incidence of BCR according to PIS in the overall population and in patients with organ confined disease and negative surgical margins (pT2R0) since they are at lower risk of recurrence [19]. Again, the same analyses were carried out for the original Irani G and A scores. All statistical tests were performed using Stata-SE 16.1 (StataCorp LP, College Station, TX, USA). Significance level was set at *p* < 0.05.

## Results

During the study period, 783 patients underwent RP; among them, 679 patients had PSA follow-up within our institution and were included in the analysis. **A total of 33 patients received adjuvant therapy (hormonal or radiation) prior to biochemical recurrence and these patients are included in the study.** The demographic and clinical characteristics of these patients are summarized in Table 1.

**Table 1:** Clinical and pathological patients’ characteristics according to novel prostatic inflammation score

	PIS 1 (*N* = 493)	PIS 2 (*N* = 123)	PIS 3 (*N* = 65)	*P* value
Age, per y	66 (62, 70)	68 (64, 72)	67 (62, 71)	**0.014**
PSA, per ng/ml	6.2 (4.6, 9.5)	5.8 (4.7, 8.2)	5.9 (4.3, 8.1)	0.3
Final GGG				
1	245 (49.7%)	52 (41.5%)	38 (58.5%)	0.8
2	104 (21.1%)	27 (22.0%)	12 (18.5%)	
3	60 (12.2%)	18 (14.6%)	7 (10.8%)	
4	59 (12.0%)	18 (14.6%)	6 (9.2%)	
5	22 (4.5%)	8 (6.5%)	2 (3.1%)	
Suspicious DRE, n (%)				
No	296 (60.0%)	57 (46.3%)	46 (70.8%)	**0.002**
Yes	197 (40.0%)	66 (53.7%)	19 (29.2%)	
Prostate volume	40 (32, 55)	40 (31, 62)	45 (40, 61)	0.053
Final GGG, n (%)				
1	171 (36.3%)	50 (42.0%)	32 (50.0%)	0.5
2	154 (32.7%)	31 (26.1%)	17 (26.6%)	
3	65 (13.8%)	13 (10.9%)	7 (10.9%)	
4	55 (11.7%)	16 (13.4%)	5 (7.8%)	
5	26 (5.5%)	9 (7.6%)	3 (4.7%)	
pT Stage				
pT2	354 (75.3%)	91 (76.5%)	56 (87.5%)	0.11
pT3a	86 (18.3%)	21 (17.6%)	3 (4.7%)	
pT3b	30 (6.4%)	7 (5.9%)	5 (7.8%)	
pR, n (%)				
Negative	305 (61.9%)	73 (59.3%)	45 (69.2%)	0.4
Positive	188 (38.1%)	50 (40.7%)	20 (30.8%)	
pN, n (%)				
Negative	215 (43.6%)	62 (50.4%)	29 (44.6%)	0.091
Positive	26 (5.3%)	12 (9.8%)	2 (3.1%)	
pNx	252 (51.1%)	49 (39.8%)	34 (52.3%)	

Bold values indicate statistical significance at p<0.05

PIS 2 and PIS 3 patients were slightly older than the PIS 1. However, no significant differences were observed in the final pathology Gleason grade groups or pT and pN stage across the PIS groups.

**Fig. 1:**
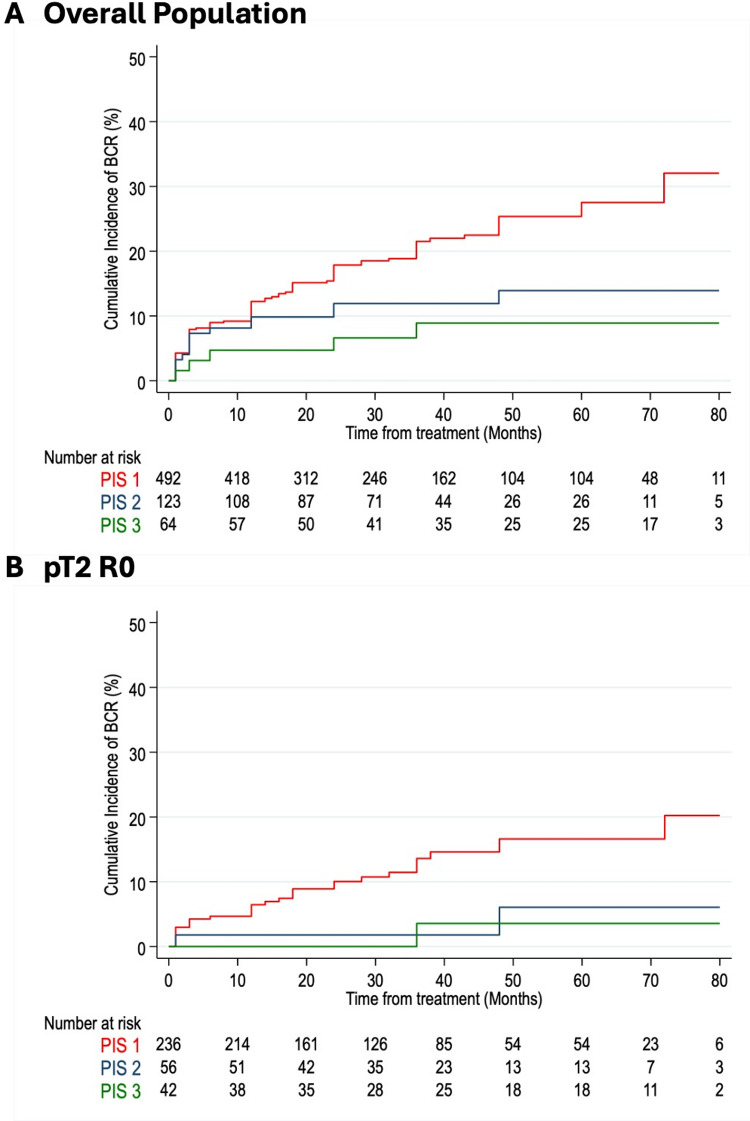
Cumulative incidence of BCR according to PIS score in the overall population (*n* = 679) and in a subgroup analysis including only patients with pT2 R0 prostate cancer (*n* = 334)

**Fig. 2:**
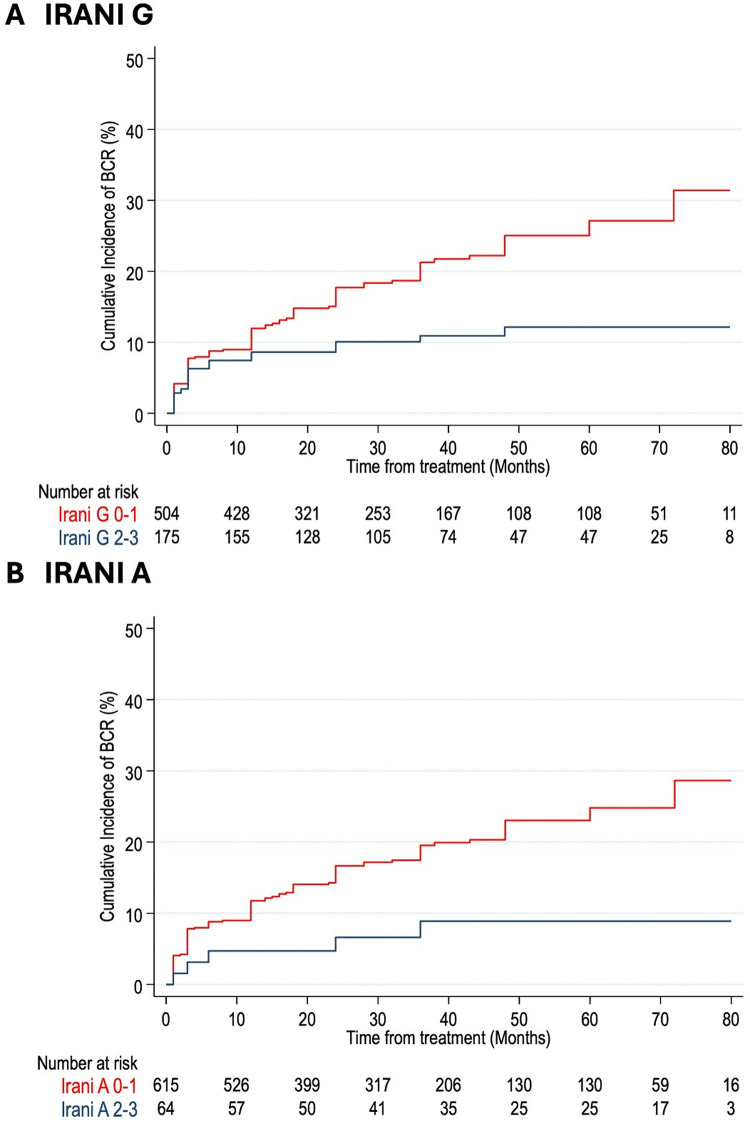
Cumulative incidence of BCR according to IRANI G and A score in the overall population (*n* = 679)

Over a median follow-up of 36.0 months (interquartile range: 18.0–60.0), 127 (18.7%) of the 679 patients experienced BCR. The cumulative incidence of BCR by PIS groups is illustrated in Fig. 1. PIS 1 patients demonstrated a significantly higher cumulative incidence of BCR (Fig. 2) compared to those PIS 2 and PIS 3, with rates of 30%, 10%, and 9%, respectively, at six years (*p* = 0.002, log-rank test). A similar trend was observed in patients with pT2R0 disease (Fig. 2).

The results of the multivariable Cox regression analysis evaluating the association of PIS score, IRANI G, and IRANI A scores with BCR are presented in Table 2. There was a significant inverse association between PIS and the risk of BCR (PIS 2: HR = 0.44, 95% CI: 0.27–0.74; PIS 3: HR = 0.37, 95% CI: 0.15–0.91). Similarly, there was a significant inverse association between IRANI G scores and the risk of BCR (HR = 0.44, 95% CI: 0.27–0.74). IRANI A scores displayed a similar trend that however did not reach statistical significance (HR = 0.41, 95% CI: 0.17–1.02; *p* = 0.054). Notably, among other adjusting covariates, pT stage and Gleason grade groups were associated with BCR while no evidence of association was shown for margin status (positive vs. negative) ad pN stage.

**Table 2:** Multivariable Cox regression analysis predicting BCR after radical prostatectomy.

IRANI G Harrell’s C = 0.7816				IRANI A Harrell’s C = 0.7732				PIS Harrell’s C = 0.7848			
ETA	HR	95% CI	*P*>|z|	ETA	HR	95% CI	*P*>|z|	ETA	HR	95% CI	*P*>|z|
Age, per y	1.00	0.97,1.03	0.779	Age,** per y**	0.99	0.96,1.02	0.494	Age, per y	1.00	0.97,1.02	0.743
PSA, per ng/ml	1.01	0.99,1.04	0.231	PSA, per ng/ml	1.02	1.00,1.04	0.109	PSA, per ng/ml	1.01	0.99,1.04	0.244
Final GGG				Final GGG				Final GGG			
1	1			1*	1			1*	Ref.		
2	1.48	0.84,2.63	0.178	2	1.59	0.90,2.81	0.110	2	1.50	0.85,2.65	0.166
3	1.58	0.79,3.17	0.195	3	1.70	0.86,3.40	0.130	3	1.60	0.80,3.19	0.185
4	1.60	0.81,3.16	0.174	4	1.71	0.87,3.36	0.118	4	1.61	0.82,3.18	0.166
5	2.69	1.27,5.66	**0.009**	5	2.47	1.17,5.22	**0.018**	5	2.65	1.26,5.59	**0.010**
pT Stage				pT Stage				pT Stage			
pT2	1			pT2	1			pT2	Ref.		
pT3a	2.37	1.51,3.71	**< 0.001**	pT3a	2.36	1.51,3.71	**< 0.001**	pT3a	2.36	1.50,3.69	**< 0.001**
pT3b	3.22	1.76,5.91	**< 0.001**	pT3b	3.54	1.95,6.43	**< 0.001**	pT3b	3.29	1.80,6.01	**< 0.001**
Margin Status				Margin Status				Margin Status			
Negative	1			Negative	1			Negative	Ref.		
Positive	1.10	0.76,1.60	0.625	Positive	1.13	0.78,1.64	0.515	Positive	1.09	0.75,1.58	0.666
PN Stage				PN Stage				PN Stage			
pN0	1			pN0	1			pN0	Ref.		
pN1	1.71	0.98,2.98	0.058	pN1	1.48	0.85,2.56	0.163	pN1	1.73	0.99,3.03	0.053
pNx	0.62	0.38,0.99	0.045	pNx	0.66	0.41,1.05	0.082	pNx	0.63	0.39,1.00	0.052
IRANI G				IRANI A				PIS			
0–1*	1			0–1*	1			1*	Ref.		
2–3	0.44	0.27,0.74	**0.002**	2–3	0.41	0.17,1.02	0.054	2	0.46	0.26,0.80	**0.007**
								3	0.37	0.15,0.91	**0.031**

*GGG* Gleason Grade group, *PIS* prostatic inflammation score

Bold values indicate statistical significance at p<0.05

## Discussion

Predicting disease outcome is a major clinical issue. This is particularly relevant for PCa, whereby factors predicting BCR are eagerly awaited, particularly for the non-negligible number of patients with favorable pathology who eventually experience unfavorable outcome in terms of early BCR.

The present study pointed out that PI can play a major role in predicting PCa outcome. Specifically, we demonstrated that the novel PIS, that joins the Irani G and A scores to overcome heterogeneity, can be used to predict BCR after RP. Kaplan-Meier analysis demonstrated a significantly higher cumulative BCR rate in PIS 1 patients compared to those in PIS 2 and PIS 3 groups. Such differences were seen also in patients with pT2R0 disease, which should be those at lower risk of BCR [19]. Multivariable analysis further highlighted that factors significantly associated with BCR were not only the well-known high stage (pT3) and high grade (GGG 5) but also PIS 1. **Diabetes also appears to play an important role**,** closely associated with prostatic inflammation**,** as observed in other studies** [12, 25].

**Pathology findings showed that biochemical recurrence (BCR) risk was highest in patients with minimal glandular and stromal inflammation (PIS 1)**,** lower in those with significant stromal but limited glandular inflammation (PIS 2)**,** and lowest in cases with high glandular inflammation**,** regardless of stromal involvement (PIS 3). These results suggest an inverse relationship between the degree/pattern of prostatic inflammation and BCR. Stromal inflammation (high Irani G) may reduce recurrence risk even with mild glandular involvement**,** while intense glandular inflammation (high Irani A) appears strongly protective.** In the last 15 years, efforts to correlate PI and PCa outcome have been mainly concentrated onto specific cells of the inflammatory infiltrate. Already in 2008, Blum et al. had pointed out that prostatic tissue expression of 3 chemokines involved in inflammatory cells recruitment was an independent predictor of BCR after RP [20]. Both CX3CL1 and CCL4 are chemokines involved in attracting immune effector cells, such as natural killer cells and T lymphocytes, although they appear to exert opposing influences on prostate cancer behavior. Indeed, CCL4 is known to have a direct proliferative and migration effects on PCa cells in vitro, whereas CX3CL1 is reported to reduce migration of PCa cells in culture [21]. Finally, also IL-15 expression, which has been suggested to prevent PCa progression by supporting NK-cell function in vivo was found to be associated with a significant reduction in BCR risk [22, 23].

Recently, it has been demonstrated that the prostatic immune content represents a significant predictor of the risk of BCR, distant metastasis and cancer specific death after RP [24]. Specifically, higher levels of mast cells, NK-cells and dendritic cells were associated with a lower risk of BCR, distant metastasis and cancer specific death, whereas higher levels of macrophages and T-cells were associated with a higher risk; B-cells and regulatory T-cells did not predict outcomes. These previous observations are consistent with the current results and support the role of inflammation as a modulator of disease progression.

**In this context**,** the integration between histological inflammation scores and transcriptomic data represents a promising tool to refine risk stratification in PCa. In our previous study**,** we demonstrated that intraprostatic chronic inflammation on biopsy was associated with adverse pathology at RP**,** even in patients with low-grade disease** [14]. **This suggests that histological inflammation may reflect a specific immune-active tumor microenvironment.**

Although assessing specific immune cells is scientifically insightful, such evaluations are resource-intensive, whereas the Irani score and PIS offer feasible alternatives during standard biopsy examination. We believe that, to keep with EAU recommendation of reporting PI in PBx cores, our findings provide grounds for routine use of the proposed PIS. If confirmed in external studies, the PIS may serve as a practical and cost-efficient tool to identify patients at increased BCR risk post-RP.

This said, our study is not without limitations. First, we did not attempt to further define site and cell type of inflammatory infiltrates, nor to compare findings in PBx with those of RP specimens, but this was beyond the scope of this study. Second, this is a retrospective analysis of data prospectively collected at a single center; therefore, external validation is eagerly awaited.

## Conclusions

The search of tools that can improve PCa risk stratification is a major clinical issue. The present study pointed out that the PIS obtained during routine pathology examination was a significant independent predictor of BCR after RP. Should our findings be externally validated, the PIS could represent a readily available inexpensive tool for predicting BCR after RP.

## Data Availability

No datasets were generated or analysed during the current study.
